# Direct costs of managing in-ward dengue patients in Sri Lanka: A prospective study

**DOI:** 10.1371/journal.pone.0258388

**Published:** 2021-10-08

**Authors:** Chathurani Sigera, Chaturaka Rodrigo, Nipun L. de Silva, Praveen Weeratunga, Deepika Fernando, Senaka Rajapakse

**Affiliations:** 1 Department of Parasitology, Faculty of Medicine, University of Colombo, Colombo, Sri Lanka; 2 Department of Pathology, School of Medical Sciences, Sydney, NSW, Australia; 3 Department of Clinical Sciences, Faculty of Medicine, Sir John Kotelawala Defence University, Ratmalana, Sri Lanka; 4 Department of Clinical Medicine, Faculty of Medicine, University of Colombo, Colombo, Sri Lanka; The University of Sydney, AUSTRALIA

## Abstract

**Introduction:**

The cost in managing hospitalised dengue patients varies across countries depending on access to healthcare, management guidelines, and state sponsored subsidies. For health budget planning, locally relevant, accurate costing data from prospective studies, is essential.

**Objective:**

To characterise the direct costs of managing hospitalised patients with suspected dengue infection in Sri Lanka.

**Methods:**

Colombo Dengue Study is a prospective single centre cohort study in Sri Lanka recruiting suspected hospitalised dengue fever patients in the first three days of fever and following them up until discharge. The diagnosis of dengue is retrospectively confirmed and the cohort therefore has a group of non-dengue fever patients with a phenotypically similar illness, managed as dengue while in hospital. The direct costs of hospital admission (base and investigation costs, excluding medication) were calculated for all recruited patients and compared between dengue and non-dengue categories as well as across subgroups (demographic, clinical or temporal) within each of these categories. We also explored if excluding dengue upfront, would lead to an overall cost saving in several hypothetical scenarios.

**Results:**

From October 2017 to February 2020, 431 adult dengue patients and 256 non-dengue fever patients were recruited. The hospitalisation costs were USD 18.02 (SD: 4.42) and USD 17.55 (SD: 4.09) per patient per day for dengue and non-dengue patients respectively (p>0.05). Laboratory investigations (haematological, biochemical and imaging) accounted for more than 50% of the total cost. The costs were largely homogenous in all subgroups within or across dengue and non-dengue categories. Excluding dengue upfront by subsidised viral genomic testing may yield overall cost savings for non-dengue patients.

**Conclusion:**

As non-dengue patients incur a similar cost per day as the dengue patients, confirming dengue diagnosis using subsidised tests for patients presenting in the first three days of fever may be cost-efficient.

## Introduction

Dengue is a global health problem with an estimated 390 million cases per year, of which majority is asymptomatic [1]. The case fatality rate of dengue is low, and in most countries, it is less than 1% [2]. However, dengue transmission is currently endemic in 129 countries and usually happens as epidemics leading to surges in-patient admissions within the space of a few weeks [2, 3]. Currently there is no robust system to triage patients at risk of developing severe dengue and hence most clinically apparent cases need close monitoring either as in-patients or as out-patients depending on the local guidelines. This monitoring incurs a significant cost to the healthcare system which is often under-appreciated [4].

Symptomatic dengue infection is an acute febrile illness usually lasting 6–10 days [5]. Some people experience complications (e.g., severe bleeding [6], pulmonary involvement [7]) resulting in a protracted course of illness [8]. Approximately 20–40% of symptomatic dengue patients have a stage of illness characterized by increased vascular permeability and extravasation of plasma, around day 5–7 since the onset of fever [9–11]. This phase, also known as the critical phase, is short lasting (48–72 hours), but if undiagnosed and mismanaged can result in shock and organ dysfunction leading to severe dengue and associated fatalities [12, 13]. Currently the onset of critical phase is identified by serial ultrasound scans or haematocrit measurements (as a surrogate measure of haemoconcentration) and these account for a large proportion of in-hospital costs of dengue management [14–18]. In addition, as patients with dengue are vulnerable to organ dysfunction (e.g., acute renal failure, hepatitis, myocarditis), physicians may also do baseline liver, renal function tests, electrocardiographs and repeat these tests when organ dysfunction is suspected [11, 13]. Confirmation of diagnosis in dengue requires either antigen testing (or RT-PCR in resourceful settings) in early infection, or immunological methods in late infection (demonstration of anti-dengue IgM or a four-fold increase in anti-dengue IgG in paired sera) [11]. All these add to the cost of patient management. If local guidelines require patients to be admitted to a hospital for monitoring (instead of being monitored as outpatients) [13], then base cost of hospital stay (e.g., food, linen, medication) also needs to be accounted for [19]. It is important that these costs are recorded and evaluated to identify how finite healthcare resources in low- and middle-income economies can be better managed without compromising quality of patient care [4].

Sri Lanka is a low-middle income country with a population of approximately 22 million and a per capita annual income of 4000 USD [20]. The per capita annual healthcare expenditure was 157 USD in 2018 (3.9% of GDP) [20]. The country is exposed to seasonal dengue epidemics coinciding with the monsoon rains and in 2017, the largest epidemic recorded since dengue surveillance began in Sri Lanka occurred with 186,101 cases and 440 deaths [21]. Diagnostic tests for dengue (NS1 antigen test, IgM test or RT-PCR) are not widely available in public health system in Sri Lanka and a large proportion of reported cases are based on a clinical case definition. All clinically suspected cases of dengue are monitored according to national guidelines on dengue management, as inpatients or outpatients [13]. A comprehensive and recent (within last 5 years) analysis of costs associated with monitoring Sri Lankan dengue patients in hospitals is not available. The last such analysis on adult dengue patients in Sri Lanka was conducted in 2012, but this dataset is limited to records of 100 adult patients. Furthermore, there was no “control” group to identify if there are additional costs involved in managing dengue compared to phenotypically similar viral fever [4].

We have been conducting a prospective cohort study (the Colombo Dengue Study—CDS) on natural history of clinically suspected dengue patients admitted to the National Hospital of Sri Lanka (Colombo) since 2017 and have built up a comprehensive database of records of clinical management [9, 22]. In all patients recruited to CDS a dengue diagnosis is confirmed by RT-PCR, but this is done retrospectively. Therefore, clinical management happens according to clinical case definition of suspected dengue (which is also the standard practice) [13]. Once dengue is confirmed retrospectively two groups of patients emerge, one with confirmed dengue and another (control) that met the clinical case definition of dengue (and managed as such) but did not have dengue fever. This design provides an ideal opportunity to study the cost effectiveness of managing dengue patients by clinical suspicion and whether investing on diagnostic testing upfront would ultimately lead to cost savings. In this study we performed an analysis of in-hospital costs (base cost of admission, and that of investigations, excluding cost of medication) associated with managing patients (with dengue or a phenotypically similar illness) with the objectives of a) describing the direct costs of managing dengue and non-dengue fever patients, b) exploring significant differences in cost across subgroups with different demographic, clinical and temporal characteristics for both dengue and non-dengue fever patients and c) comparing several hypothetical scenarios to see if excluding dengue upfront would ultimately result in cost savings for non-dengue fever patients due to less hospital admissions.

## Methods

The Colombo Dengue Study (CDS) is an ongoing prospective cohort study that recruits symptomatic dengue patients admitted to the National Hospital of Sri Lanka (NHSL) in Colombo. CDS is conducted in two phases and Phase 1 was conducted between October 2017 and February 2020 (this analysis is for Phase 1 only). CDS aims to record natural history of dengue fever and build a comprehensive predictive model to identify people at risk of plasma leakage, early in the infection. The details of this collaborative project led by investigators of University of Colombo, Sri Lanka and University of New South Wales, Australia has been published previously [9, 22]. In brief, CDS recruits’ adults clinically suspected of having dengue fever (as evaluated by two independent medical officers to meet the clinical case definition of dengue [13]) admitted to NHSL within the first three days of fever. Diagnosis of dengue is confirmed by either an NS1 antigen test (one step SD Bioline dengue NS 1 antigen test, Alere SD, USA) or an RT-PCR [23]. Any patients with a confirmed alternative diagnosis during hospital stay are excluded (in these patients, dengue co-infection was excluded with an NS1 antigen test). Since RT-PCR for dengue is done by batch processing (due to logistical reasons), confirmation of diagnosis by this test is retrospective and does not bias the in-patient management or decision making. Patients who were negative for both tests (NS1 test and RT-PCR) are retrospectively assigned as non-dengue fever (NDF) patients, but they were clinically managed as dengue patients while in hospital. This replicates the real-world management of dengue patients as RT-PCR is not available as a routine diagnostic test (except for research purposes) in the public health sector to confirm dengue infection. Patients were interviewed on admission to record their demographic, socioeconomic and clinical sign / symptom profile and were followed up daily by the same investigator to maintain a detailed clinical record of evolution of illness (severe vs. non severe dengue fever, presence or absence of plasma leakage) and the investigations performed. Severe dengue was defined as in the 2009 WHO guidelines [11]. Once the patient was discharged, the outcome was recorded. The curated dataset (a record per patient) is maintained in REDCap (Version 9.1, Vanderbilt University, USA) in a secure access server hosted by the University of Colombo, Sri Lanka.

For the analysis presented in this paper, we extracted the medical investigation profile per patient (haematological, microbiological, biochemical tests and imaging) and the duration of hospital stay. The costs for each investigation were extracted from prescribed costs for public health sector institutes by the Ministry of Health in Sri Lanka (last revised in 3.02.2020 and 01.09.2017) [19, 24]. This analysis considers the cost from the government perspective and not from patient perspective. Hence even if a patient had a test done from a private healthcare provider, for purposes of analysis, it was considered as done in the public health sector. This allowed standardization of costs as the prices are invariably marked up in the private healthcare sector (and varies across different healthcare providers) to include a higher margin of profit. Sri Lanka has a universal public health system that provides in-patient services and investigations free of charge to all citizens and therefore most costs for patients in our cohort were paid by the taxpayer. All tests typically performed for a dengue patient are available in National Hospital of Sri Lanka. The baseline cost of hospital stay (e.g., bed, linen, food, service charges and utilities) was also extracted from the cost schedule for Ministry of Health. Dengue has no specific treatment, and most patients receive antipyretics and intravenous fluids only, as required. Hence the medication cost is expected to be low, compare to the investigation cost, unless the patient is admitted to an intensive care unit. The government circular from which the unit costs were extracted, does not list costs of individual medicines but recommends a flat rate for all patients (LKR 500, USD 2.7) per day. In our opinion including this rate will be an overestimate for dengue, and hence cost of medication was excluded from this analysis.

The sample size calculation for CDS has been published previously [9, 22] and this costing study includes all patients recruited to CDS by February 2020 (Phase 1 of CDS). All costs are indicated in United States dollars (USD) in the main manuscript and in Sri Lankan Rupees (LKR) in the S1–S5 Tables, using the average exchange rate of one United States dollar to 185.43 LKR in 2020. The costs are also classified according to demographic (gender, age), temporal (month of admission, 3-month intervals within a calendar year) and clinical (severe dengue, infecting serotype, presence of plasma leakage, presence of metabolic comorbidities) subgroups and compared across these subgroups within the DF and NDF categories. Since investigations account for most of the cost variation per patient, the total and individual costs for investigations are given separately for DF and NDF patients. Descriptive statistics are shown with a base unit of cost of USD per patient per day with standard deviation (SD). Significant differences across categories were explored an independent T- test. A p value < 0.05 was considered statistically significant. For multiple comparisons, the p value was adjusted using the Bonferroni correction.

We also explored if having a diagnosis of dengue upfront will reduce the overall costs for NDF patients. The clinical scenarios for NDF patients (if dengue was excluded) are complex as not all patients may be discharged home despite a negative test. CDS recruits patients in the first 3 days of fever, where a RT-PCR and NS1 test is highly likely to be positive due to early viraemia if the patient has dengue. However, in severely ill patients meeting the clinical case definition of dengue, even if the RT-PCR were negative it is likely that they would be re-tested with a dengue IgM antibody test after day 5 of illness (when the first antibody response appears against dengue) and therefore may remain admitted in hospital and managed as a dengue patient to err on the side of caution. Similarly, less severely ill patients may be sent home but may come back few days later for a dengue IgM antibody test if their symptoms do not resolve. It is difficult to predict what this percentage of returning patients would be without real-life data from a clinical trial, and therefore our model was built as follows.

We calculated a symptom score for all NDF patients on admission giving equal weighting to all of the following symptoms (a score of 1 if present, range 0–13); abdominal pain, arthralgia, cough, chills or rigors, difficulty in breathing, headache, fainting, loss of appetite, backache, myalgia, nausea or vomiting, retro-orbital pain, vertigo (fever is not in the score as all patients must have had fever to be included in CDS). NDF Patients were divided into quartiles based on the symptom score and we assumed patients in Q1 (less severe symptoms on admission) will have a full blood count, NS1 test and a RT-PCR on admission, will be discharged home after excluding dengue and will not return to hospital. It was assumed that patients in Q2 and Q3 would receive the same diagnostic tests on admission as Q1, will be discharged home, but a proportion will return for a dengue IgM antibody test if their symptoms do not subside. We predicted the costs in three scenarios based on this returning percentage being 0%, 20% or 50%. Assumptions for patients in Q4 were that they will receive the same tests on admission as others but will not be discharged home, will receive the same care that they had received in real-life (treated as a dengue positive patient, to err on the side of caution). We then calculated costs for all scenarios separately, summed up the costs for all quartiles and calculated the average cost of per NDF patient to compare with the actual costs incurred per NDF patient when an upfront diagnosis was not available.

When including the cost of RT-PCR for the above purpose, there was no unit cost in the government schedule as this test is not offered in the public health sector. Based on our reagent cost (with a 10% markup for labour and utilities), the cost was USD 22.4 (for diagnosis of dengue only, excluding serotyping). However, quotes from three main private health care providers in Colombo (in April 2021) averaged at USD 65.6. Given this huge discrepancy, we calculated costs based on both (USD 22.4 and 65.6) prices. The cost of dengue IgM antibody test was approximately USD 4.0.

The data reported in this paper are covered by the ethics approval for Colombo Dengue Study from the Ethics Review Committee of University of Colombo, Sri Lanka (EC/17/080) and the National Hospital of Sri Lanka (ETH/COM/2017/12).

## Results

**Fig 1:**
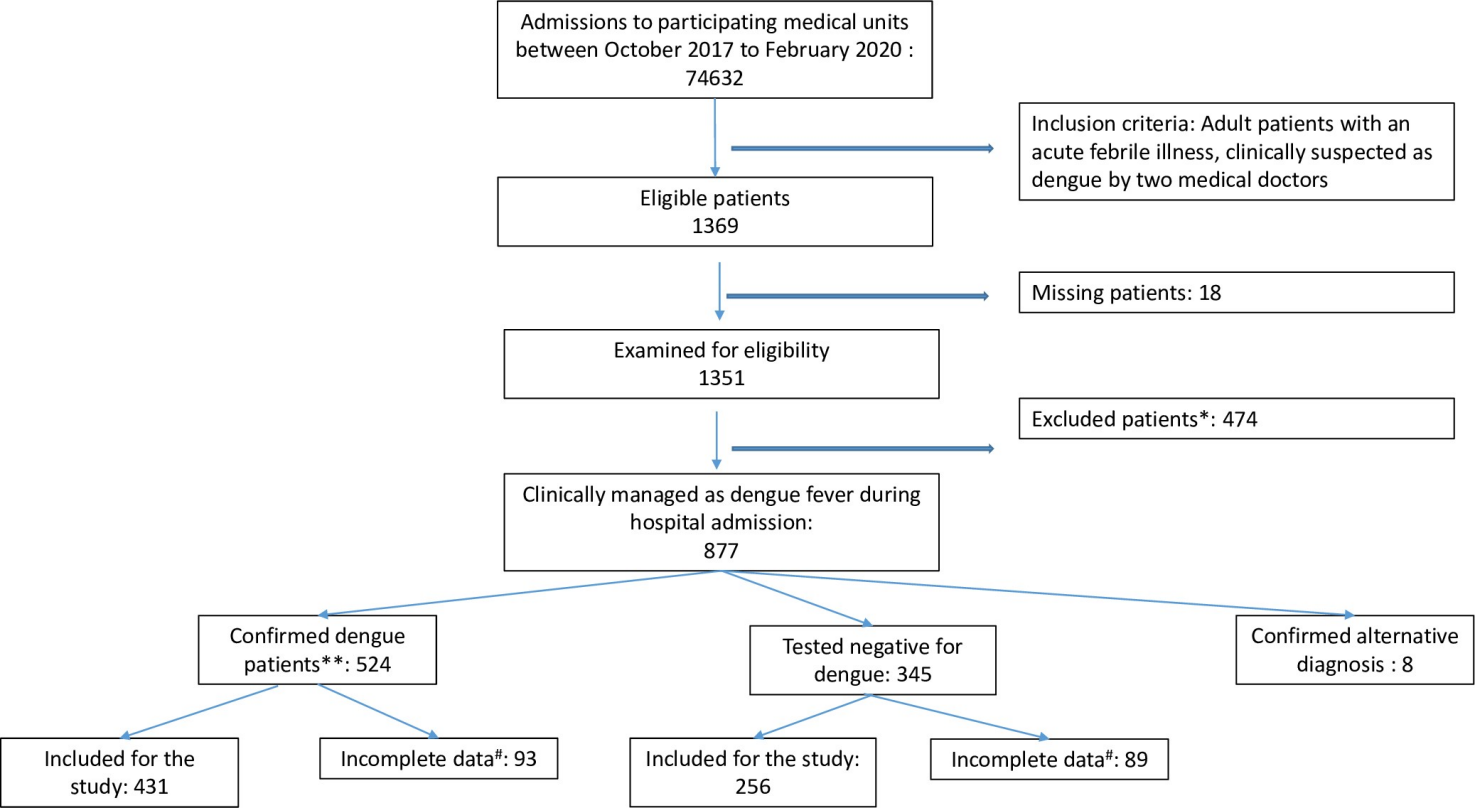
Flowchart showing patient recruitment to Colombo Dengue Study.

A total of 869 patients (DF: 524, NDF: 345) were recruited to Phase 1 of CDS between October 2017 and February 2020. However, data was incomplete for 93 DF and 89 NDF patients. This analysis includes 431 (males– 285, 66.1%, mean age: 31.4 years) confirmed DF patients and 256 NDF patients (males –176, 68.8%, mean age: 37.7 years) who had complete data records. Flow chart of patients recruited to this study is shown in Fig 1. The mean duration of hospital stay was 3.94 (SD:+/-1.68) and 2.84 (SD: +/-1.54) days for DF and NDF patients respectively. All patients were discharged alive after resolution of the illness and none were admitted to an intensive care unit. The average cost of hospitalisation (base cost of hospital stay plus investigations) was USD 18.02 (SD: +/-4.42) per patient per day for DF patients and USD 17.55 (SD: +/-4.09) per patient per day for NDF patients. On average, this amounts to USD 67.87 (SD: +/-25.77) per stay for dengue patients and USD 47.36 (SD: +/-21.60) per stay for non-dengue patients. There was no significant difference of the standardised daily cost between these groups but as the dengue patients had a longer stay in hospital, the average cost per total stay was significantly higher for them (p<0.05). Within this average total cost, 55.1% and 53.8% were spent on investigations for DF and NDF patients respectively. The total direct cost of hospitalization (excluding medication costs) for all patients in the study was USD 29,307.87 for DF and USD 12,123.72 for NDF patients (actual cost, not averages).

A further breakdown of the cost based on clinical, temporal and demographic characteristics of the cohort is shown in Table 1 (see S1 Table for LKR values). On average, females incurred a higher total cost (per patient per day) than males. When age groups were considered, patients > 70 years of age incurred the highest cost in DF patients. People with metabolic comorbidities had a lower cost compared to those without, while those with non-severe dengue (vs. severe dengue) or with serotype 2 infections (vs. other serotypes combined) also incurred a lower cost. However, when mean differences were compared between these groupings within DF and NDF categories or across similar groupings in DF and NDF patients, none of these differences were statistically significant (Tables 2 and 3 in USD values, also see S2 and S3 Tables for LKR values).

**Table 1:** Standardised costs (as cost per patient per day) for dengue fever (DF) and non-dengue fever (NDF) patients in Colombo Dengue Study.

Characteristic	Dengue fever			Non-dengue fever		
	Number of patients	Total cost in USD (SD)	Cost of investigations in USD (SD)	Number of patients	Total cost in USD (SD)	Cost of investigations in USD (SD)
All patients	431	18.02 (4.42)	9.93 (4.42)	256	17.52 (4.09)	9.43 (4.09)
*Gender*						
Male	285	17.90 (4.17)	9.81 (4.17)	176	17.60 (4.23)	9.51 (4.23)
Female	146	18.26 (4.88)	10.17 (4.88)	80	17.34 (3.80)	9.25 (3.80)
*Age group*						
< = 20 years	97	18.02 (4.07)	9.92 (4.07)	44	17.89 (3.60)	9.80 (3.60)
21–30 years	161	18.06 (4.67)	9.97 (4.67)	58	17.79 (5.19)	9.70 (5.18)
31–40 years	73	18.23 (4.78)	10.14 (4.78)	50	17.31 (3.18)	9.21 (3.18)
41–50 years	50	17.59 (3.89)	9.50 (3.89)	41	16.83 (4.57)	8.74 (4.57)
51–60 years	34	18.51 (4.56)	10.42 (4.56)	31	17.80 (4.29)	9.71 (4.29)
61–70 years	13	16.93 (3.97)	8.84 (3.97)	23	17.44 (3.07)	9.34 (3.07)
> = 71 years	2	19.61 (3.91)	11.52 (3.91)	9	17.52 (2.61)	9.43 (2.61)
*Metabolic comorbidities*						
Yes	76	17.93 (4.08)	9.84 (4.08)	70	16.95 (3.04)	8.86 (3.04)
No	355	18.04 (4.50)	9.95 (4.50)	186	7.73 (4.41)	9.64 (4.41)
*Plasma leakage*						
Yes	132	17.87 (3.89)	9.78 (3.89)	NA	NA	NA
No	299	18.09 (4.64)	10.00 (4.64)	NA	NA	NA
*Severe dengue*						
Yes	25	18.46 (5.35)	10.37 (5.35)	NA	NA	NA
No	406	17.99 (4.37)	9.90 (4.37)	NA	NA	NA
*Serotype*						
DENV-2	229	18.05 (4.94)	9.96 (4.94)	NA	NA	NA
Others	120	18.14 (3.86)	10.05 (3.86)	NA	NA	NA
*Month of admission*						
January	47	17.59 (3.44)	9.50 (3.44)	23	16.64 (3.53)	8.55 (3.53)
February	16	16.80 (3.73)	8.71 (3.73)	12	18.62 (4.31)	10.53 (4.31)
March	20	18.67 (8.60)	10.58 (8.60)	19	17.85 (3.53)	9.76 (3.53)
April	27	17.83 (4.86)	9.74 (4.86)	14	17.22 (3.26)	9.13 (3.26)
May	19	17.13 (3.38)	9.04 (3.38)	20	16.34 (2.39)	8.25 (2.39)
June	58	17.90 (4.75)	9.81 (4.75)	47	17.58 (4.09)	9.49 (4.09)
July	44	16.90 (2.52)	8.81 (2.52)	27	16.86 (3.60)	8.77 (3.60)
August	21	19.48 (6.17)	11.38 (6.17)	14	17.48 (9.42)	9.39 (9.42)
September	28	18.41 (4.02)	10.32 (4.02)	16	15.99 (2.24)	7.89 (2.24)
October	36	18.43 (3.45)	10.33 (3.45)	23	18.31 (3.82)	10.22 (3.82)
November	57	18.90 (4.67)	10.81 (4.67)	25	19.28 (2.96)	11.19 (2.96)
December	58	18.01 (3.82)	9.92 (3.82)	16	17.89 (4.05)	9.80 (4.05)
*Timing of admission (3-month windows)*						
2017 (October to December)	27	15.95 (2.50)	7.86 (2.50)	2	16.05 (0.57)	7.96 (0.57)
2018 (January to March)	30	18.27 (7.31)	10.18 (7.31)	16	17.43 (3.75)	9.34 (3.75)
2018 (April to June)	90	17.83 (4.69)	9.74 (4.69)	67	17.03 (3.60)	8.94 (3.60)
2018 (July to September)	45	18.67 (5.13)	10.58 (5.13)	35	16.19 (3.53)	8.09 (3.53)
2018 (October to December)	49	17.91 (3.32)	9.82 (3.32)	18	17.18 (3.74)	9.09 (3.74)
2019 (January to March)	21	17.07 (2.97)	8.98 (2.97)	10	16.63 (0.57)	8.54 (0.57)
2019 (April to June)	16	17.31 (3.33)	9.22 (3.33)	16	18.16 (3.49)	10.07 (3.49)
2019 (July to September)	46	17.20 (2.82)	9.11 (2.82)	20	17.60 (7.66)	9.51 (7.66)
2019 (October to December)	75	19.69 (4.51)	11.59 (4.51)	44	19.28 (3.38)	11.19 (3.38)
2020 (January to February)	32	17.58 (3.79)	9.49 (3.79)	28	17.86 (4.00)	9.77 (4.00)

Footnote: 1 USD = 185.40 LKR as per average exchange rate for 2020, Unit of costing–Cost per patient per day, Metabolic comorbidities include diabetes mellitus, hypertension, hyperlipidaemia, past history of major cardiovascular events or a combination of these.

**Table 2:** Mean differences of costs within DF and NDF patient groups (the first subgroup in each category is the comparator, unit–cost per patient per day in USD).

Characteristic	Dengue fever		Non-dengue fever	
	Mean difference in USD	P value	Mean difference in USD	P value
Gender				
Male	-	-	-	-
Female	-0.37	0.4160	0.26	0.6389
Age group				
Age< = 20 years	-	-	-	-
Age 21–30 years	-0.05	0.9362	0.10	0.9146
Age 31–40 years	-0.22	0.7487	0.58	0.4054
Age 41–50 years	0.42	0.5447	1.06	0.2380
Age 51–60 years	-0.49	0.5556	0.09	0.9216
Age 61–70 years	1.08	0.3683	0.45	0.6088
Age > = 71 years	-1.60	0.5833	0.37	0.7696
Metabolic comorbidities				
Yes	-	-	-	-
No	-0.11	0.8434	-0.78	0.1746
Plasma leakage				
Yes	-	-	-	-
No	-0.21	0.6447	NA	NA
Severe dengue				
Yes	-	-	-	-
No	0.46	0.6112	NA	NA
Serotype				
DENV-2	-	-	-	-
Others	-0.09	0.8607	NA	NA
Month of admission				
January	-	-	-	-
February	0.80	0.4360	-1.98	0.1532
March	-1.07	0.4650	-1.21	0.2752
April	-0.23	0.8100	-0.58	0.6206
May	0.46	0.6207	0.30	0.7525
June	-0.31	0.7122	-0.95	0.3463
July	0.69	0.2789	-0.22	0.8261
August	-1.88	0.1119	-0.85	0.6976
September	-0.81	0.3548	0.65	0.5194
October	-0.83	0.2790	-1.67	0.1299
November	-1.30	0.1155	-2.65	0.0070*
December	-0.41	0.5661	-1.26	0.3093
Year of admission				
2018	-	-		
2019	-0.29	0.5497	-1.53	0.0060*

Reference category for comparison, Unit of costing–cost per patient per day

* Statistically significant with the p value of <0.05

** Statistically significant with the Bonferroni adjusted p value of <0.007

**Table 3:** Mean differences of costs across DF and NDF patient groups (a positive mean difference indicates a higher cost in DF group, unit–cost per patient per day in USD).

Characteristic	Dengue and Non-dengue fever	
	Mean difference in USD	P value
Gender		
Male	0.30	0.4610
Female	0.92	0.1440
Age group		
Age< = 20 years	0.13	0.8603
Age 21–30 years	0.27	0.7142
Age 31–40 years	0.93	0.2315
Age 41–50 years	0.76	0.3942
Age 51–60 years	0.71	0.5208
Age 61–70 years	0.50	0.6738
Age > = 71 years	2.10	0.3600
Metabolic comorbidities		
Yes	0.98	0.1056
No	0.31	0.4461
Month of admission		
January	0.96	0.2820
February	-1.82	0.2417
March	0.82	0.7018
April	0.61	0.6757
May	0.79	0.4022
June	0.32	0.7184
July	0.04	0.9547
August	1.99	0.4541
September	2.42	0.0322*
October	0.11	0.9054
November	0.39	0.7028
December	0.11	0.9180
Year of admission		
2017 (October to December)	0.09	0.9584
2018 (January to March)	0.84	0.6716
2018 (April to June)	0.80	0.2453
2018 (July to September)	2.48	0.0166*
2018 (October to December)	0.74	0.4393
2019 (January to March)	0.44	0.7041
2019 (April to June)	-0.85	0.4875
2019 (July to September)	- 0.40	0.7564
2019 (October to December)	0.41	0.6061
2020 (January to February)	-0.28	0.7836

* Statistically significant with the p value of <0.05

** Statistically significant with the Bonferroni adjusted p value of <0.004 (month of admission), p value of <0.005 (year of admission)

A large proportion of costs was for investigations. In terms of absolute numbers, Full Blood Count, AST/ALT and serum electrolytes were the most frequently performed investigations in that order and in terms of cost, most money had been spent on doing full blood counts, NS 1 antigen tests and ultrasound scans. A detailed breakdown of number of investigations and cumulative costs are given in Table 4 (see S4 Table for LKR values).

**Table 4:** Investigation numbers and associated costs during the 29-month assessment period.

Investigation	Cost per unit in USD	Number of tests	Total investigation cost in USD
Full blood count	1.35	5840	7874.87
AST	0.54	1742	939.59
ALT	0.54	1742	939.59
Serum electrolyte	1.35	1488	2006.47
Serum creatinine	0.81	1476	1194.17
CRP	1.08	1311	1414.24
Ultrasound scan	2.43	1085	2633.50
Bilirubin	0.81	781	631.88
NS 1 test	4.21	687	2890.29
UFR	0.49	201	97.57
Creatine Kinase (CPK)	1.62	203	328.48
ALP	0.81	137	110.84
ESR	0.27	111	29.94
X-rays	0.81	103	83.33
ECG	0.27	87	23.46
PT/INR	0.54	81	43.69
Albumin and globulin	0.67	69	46.52
Serum protein	0.67	63	42.48
APTT	0.81	58	46.93
Urine culture + ABST	1.89	35	66.07
Amylase	1.08	35	37.76
Serum urea	0.54	34	18.34
Gamma GT	0.81	29	23.46
Serum calcium	0.81	27	21.84
Blood culture +ABST	1.89	24	45.31
Plasma glucose	0.67	10	6.74
Troponin	4.31	9	38.83
Sputum culture + ABST	1.89	8	15.10
Echocardiography	10.79	7	75.51
IgG test	2.16	5	10.79
IgM test	2.16	4	8.63
Stool culture +ABST	1.89	3	5.66
Total cholesterol	0.81	3	2.43

Footnote: ABST: Antibiotic Sensitivity Test, ALP: Alkaline Phosphatase, ALT: Alanine Aminotransferase, APTT: Activated Partial Thromboplastin Time, AST: Aspartate Aminotransferase, CRP: C Reactive Protein, ECG: Electrocardiogram, ESR: Erythrocyte Sedimentation Rate, Gamma GT: Gamma Glutamyl Transferase, IgG: Immunoglobulin G, IgM: Immunoglobulin M, INR: International Normalized Ratio, NS1: Non-Structural protein 1, PT: Prothrombin Time, UFR: Urine Full Report

When costs were recalculated in hypothetical scenarios to see if excluding dengue upfront will be cost saving for NDF patients, if the cost of RT-PCR is USD 22.4, in all scenarios considered, (0%, 20% and 50% of Q2 and Q3 patients returning for an IgM test) the total cost per patient was still cheaper (USD 35.73–36.53) than the observed average cost per stay for NDF patients (USD 47.36). However, if the cost of RT-PCR is USD 65.6, then all the hypothetical scenarios were more expensive (USD 78.9–79.7) than the actual cost (S5 Table).

## Discussion

This prospective study of 431 confirmed adult DF patients and parallelly recruited 256 NDF patients (who had a phenotypically similar illness), places the direct cost of in-hospital care (base cost of admission and investigation cost, excluding cost of medication) to be around USD 18 per patient per day. This cost was largely homogenous across Df and NDF categories, different age and gender groups, groups with and without metabolic comorbidities, month of admission, regardless of viral serotype and whether a critical phase was observed or not, during the illness. If dengue was excluded upfront by an RT-PCR offered at a subsidised rate, cost savings are likely due to less hospital admissions in the NDF category.

The hospitalization costs of dengue are unlikely to be uniformly distributed across endemic countries as these are influenced by local guidelines for diagnosis and management, access to healthcare, differential costing between public and private sector healthcare providers and total available healthcare budget. Therefore, it is essential that reliable local data are published on healthcare costs to assist in future planning. In Sri Lanka as mentioned above, the annual healthcare allocation in the budget is around 3.9% of its gross domestic product (GDP). In 2019, this allocation was LKR 185.5 billion (USD 1 billion) [25]. In the same year, Sri Lanka reported a total of 105,049 suspected dengue cases [26] and assuming 37.2% of these would-be non-dengue fever (based on observations reported above), a crude extrapolation places the total cost of hospitalisation and investigating at USD 6.55 million (0.7% of annual healthcare allocation for 2019) for all clinically suspected dengue patients. Healthcare in Sri Lanka is provided through both state (public) institutions and private institutions. For Sri Lankan citizens, services in public hospitals are completely free and paid by the state. However, this leads to overcrowding and waiting lists for some procedures and this is made worse during seasonal dengue epidemics as hospitals are overcrowded with fever patients that needs close monitoring. Thus, the actual cost of providing health care for these patients needs to be carefully scrutinised for cost effectiveness.

In subgroup comparisons, we expected to find age and/or gender-based differences or differences based on infecting serotype as such differences were reported in literature in other countries (see below). We also compared costs for each month of the year assuming that these would be higher at times of epidemics (November–February and May to September, coinciding with monsoon rain seasons) [21] or when a new rotation of relatively inexperienced junior doctors takeover (in January and July). Under these circumstances more patients may be managed as dengue, erring on the side of caution, when confirmatory diagnostic tests are not always performed. We also compared two different years as some years have a worse epidemic than others (51,659 reported cases in 2018 vs. 105,049 in 2019) [26]. The cost comparison between DF and NDF groups is important as diagnosis of dengue is usually not confirmed in public health sector in Sri Lanka and instead, a clinical case definition is used to manage patients. Though we confirmed dengue using RT-PCR in this study, this information was not available to the treating physicians when the patient was in hospital. Yet, the costs were largely homogenous across DF and NDF categories and across almost all subgroups considered, and this can be interpreted in several ways. It is reassuring that at least in this sample there were no subgroup-based cost differences probably due to the strict adherence to clinical management guidelines. This also indicates that cost is not influenced by healthcare worker rotations or by the intensity of an ongoing epidemic. However, this also raises the question whether costs would be less if the diagnosis of NDF was available to the treating physicians upfront. If so, these patients may have been discharged or observed less intensely and this would have manifested as statistically significant reduction of daily costs. The cost modelling in a range of hypothetical scenarios to see if this assumption had merit, revealed that this will depend on the cost of a RT-PCR. If the government can establish facilities and offer this test at a subsidised rate, it may be cost saving on the long run. However, we cannot speculate on the capital costs in establishing such laboratory facilities and this were not considered in the costing. On the other hand, if the RT-PCR was to be done at commercial rates, then the current practice of treating all NDF patient as dengue patients, without confirming the diagnosis, is still safe and a cheaper option.

The economic cost of dengue management in hospitals is not trivial. A systematic review of studies from 18 countries (adjusted for exchange rates in 2015), showed that the direct and indirect costs of dengue infection in these countries to account for USD 3.3 billion purchasing power parity (a theoretical exchange rate that can “buy the same basket of goods” in each country) [27]. In Sri Lanka, a previous analysis (in 2012) placed the cost of hospitalization of an adult dengue patient at USD 196–866 and that for a paediatric patient at USD 216–609 for the total duration of stay [4]. However, this was a retrospective analysis of data items extracted from only 100 hospital records, randomly selected from three hospitals in the Colombo district in Sri Lanka in a single year. This study also had ICU admitted patients explaining for the high variation in cost but did not have a “control” group to identify if there are any additional expenses associated with dengue fever compared to phenotypically similar viral fever. This previous study also lacks a comprehensive subgroup analysis as presented here, probably due to the smaller sample size. In another single centre prospective analysis from Colombo, Sri Lanka in 2012/13 with 130 paediatric patients, the average cost of a hospital stay was estimated to be USD 80 for dengue fever and USD 191 for dengue haemorrhagic fever (average hospital stay: 3.8 and 4.8 days respectively) [28]. Our study is comparable with this analysis as we excluded paediatric patients.

There are no other recent similar studies from Sri Lanka but those from other Southeast Asian and Latin American countries show the costs to be highly variable (partly confounded by sample size variations and variation in study designs) in each country. In Brazil a cross sectional analysis of 288 dengue patients admitted to hospitals in 2010 (records analysed retrospectively) revealed an average cost of USD 260 per stay (range: 179–621, mean length of stay: 4.3 days) [29]. The costs were significantly higher for patients older than 60 years compared to children < 15 years (USD 201 vs. 382, p<0.05). However, this observation may have been confounded by the small sample size, recall bias of a retrospective analysis and non-standardization of unit costs due to sampling across public and private health care providers. A retrospective analysis from China with a larger sample size (n = 1432) in 2013–2014 showed a higher cost per patient per stay (USD 500) but the average duration of stay was also longer (7.2 days) [30]. This study also demonstrated a higher cost for people aged > 65 years compared to paediatric patients (< 14 years). In Thailand, a prospective study of 224 dengue patients in public hospitals in 2015 shows an average cost of USD 82 and 109 per paediatric and adult patient per stay, respectively (average duration of stay: 3.9 days) [31]. Seven studies from Vietnam place the direct cost of dengue hospital admissions from Vietnam in the range of USD 88–214 per admission [32, 33]. Closer to Sri Lanka, data from a single centre in South India shows the median hospitalization cost of an adult dengue patient (n = 50, median duration of stay: 5 days) to be USD 317 for non-severe dengue and USD 720 for severe dengue [34].

Compared to the previous costing study reported from Sri Lanka for adult dengue patients [4], this study has methodological strengths of using a larger cohort, prospectively recruiting and following up patients which allowed reliable record keeping and calculation of actual costs rather than estimates based on the scrutiny of randomly selected hospital records retrospectively. In addition, this study had a comparator group of NDF patients with a phenotypically similar illness. Even within the DF group we had multiple subgroups based on disease severity. This included severe and non-severe dengue subgroups as per 2009 WHO guidelines [11], as well as subgroups based on presence or absence of plasma leakage. In identifying costs per item, we relied on numbers provided for public sector hospitals by the Ministry of Health or the Medical Research Institute which is under the same Ministry. This ensured that the indicated costs are close to the base cost (the margin of profit is much higher with private healthcare services) for providing such services in Sri Lanka.

This study also has several limitations in that this is a single centre study, which excluded paediatric patients. Hence the generalizability of results needs to be considered within this context. NHSL is a well-staffed teaching hospital with 24-hour laboratory and imaging services. For smaller peripheral hospitals, we assume the costs per patient would be less due to non-availability or reduced accessibility to some of the investigations. On the other hand, this may prove to be costly in some instances as missed opportunities to intervene may culminate in a complication requiring intensive care or transfer to a higher tier hospital. None of the patients in CDS were admitted to an ICU but the inclusion criteria of CDS (patients admitted in the first three days of fever before plasma leakage) biases against ICU admission which is more likely to be seen with late presentations or for those transferred from other hospitals with complications. Inclusion of ICU patients would have increased the estimated cost per patient significantly as shown in previous literature [4]. Finally, the cost calculations presented here excludes the cost of medication and the reason for this is explained in the methods.

## Conclusion

This single centre prospective study conducted over two and half years (2017–2020) showed that the average daily cost of managing hospitalized DF and NDF patients (with a phenotypically similar illness) to be the same. The daily costs were also largely homogenous across different subgroups within the DF and NDF categories. However, as dengue patients stayed longer in the hospital, the cost per stay was significantly higher in this group. Exclusion of dengue in patients presenting within the first three days of fever, by a subsidised RT-PCR can be cost saving in the long-term. There is an opportunity to explore this further, ideally as a clinical trial.

## Supporting information

S1 Table Standardised costs (as cost per patient per day in LKR) for dengue fever (DF) and non-dengue fever (NDF) patients in Colombo Dengue Study. (DOCX)

S2 Table Mean differences of costs within DF and NDF patient groups (the first subgroup in each category is the comparator, unit–cost per patient per day in LKR). (DOCX)

S3 Table Mean differences of costs across DF and NDF patient groups (a positive mean difference indicates a higher cost in DF group, unit–cost per patient per day in LKR). (DOCX)

S4 Table Investigation numbers and associated costs in LKR during the 29-month assessment period. (DOCX)

S5 Table Recalculated cost for non-dengue fever patients based on several hypothetical scenarios assuming RT-PCR based exclusion of dengue diagnosis was available on admission. (DOCX)

S1 Checklist STROBE checklist for this manuscript. (PDF)
