# Supplementary material for: Direct costs of managing in-ward dengue patients in Sri Lanka: A prospective study
Source: PLoS One. 2021 Oct 8;16(10):e0258388. doi: 10.1371/journal.pone.0258388 (PMC8500425; doi:10.1371/journal.pone.0258388)
Supplement: S1 Table — (DOCX) [file pone.0258388.s001.docx]

**Supplementary Table 1**. Standardised costs (as cost per patient per day in LKR) for dengue fever (DF) and non-dengue fever (NDF) patients in Colombo Dengue Study

| Characteristic | Dengue fever | | | Non-dengue fever | | |
| --- | --- | --- | --- | --- | --- | --- |
|  | Number of patients | Total cost in LKR (SD) | Cost of investigations in LKR (SD) | Number of patients | Total cost in LKR (SD) | Cost of investigations in LKR (SD) |
| All patients | 431 | 3341 (820) | 1841 (820) | 256 | 3248 (759) | 1748 (759) |
| *Gender* |  |  |  |  |  |  |
| Male | 285 | 3318 (774) | 1818 (774) | 176 | 3263 (784) | 1763 (784) |
| Female | 146 | 3386 (904) | 1886 (904) | 80 | 3215 (704) | 1715 (704) |
| *Age group* |  |  |  |  |  |  |
| <=20 years | 97 | 3340 (754) | 1840 (754) | 44 | 3317 (667) | 1817 (667) |
| 21-30 years | 161 | 3348 (865) | 1848 (865) | 58 | 3298 (962) | 1798 (962) |
| 31-40 years | 73 | 3380 (886) | 1880 (886) | 50 | 3208 (589) | 1708 (589) |
| 41-50 years | 50 | 3261 (721) | 1761 (721) | 41 | 3121 (848) | 1621 (848) |
| 51-60 years | 34 | 3432 (845) | 1932 (845) | 31 | 3300 (795) | 1800 (795) |
| 61-70 years | 13 | 3139 (736) | 1639 (736) | 23 | 3233 (569) | 1733 (569) |
| >=71 years | 2 | 3636 (724) | 2136 (724) | 9 | 3247 (484) | 1747 (484) |
| *Metabolic comorbidities* |  |  |  |  |  |  |
| Yes | 76 | 3324 (757) | 1824 (757) | 70 | 3143 (564) | 1643 (564) |
| No | 355 | 3345 (834) | 1845 (834) | 186 | 3287 (818) | 1787 (818) |
| *Plasma leakage* |  |  |  |  |  |  |
| Yes | 132 | 3314 (721) | 1814 (721) | NA | NA | NA |
| No | 299 | 3353 (861) | 1853 (861) | NA | NA | NA |
| *Severe dengue* |  |  |  |  |  |  |
| Yes | 25 | 3422 (991) | 1922 (991) | NA | NA | NA |
| No | 406 | 3336 (810) | 1836 (810) | NA | NA | NA |
| *Serotype* |  |  |  |  |  |  |
| DENV-2 | 229 | 3346 (916) | 1846 (916) | NA | NA | NA |
| Others | 120 | 3363 (715) | 1863 (715) | NA | NA | NA |
| *Month of admission* |  |  |  |  |  |  |
| January | 47 | 3262 (638) | 1762 (638) | 23 | 3084 (655) | 1584 (655) |
| February | 16 | 3114 (692) | 1614 (692) | 12 | 3452 (799) | 1952 (799) |
| March | 20 | 3461 (1594) | 1961 (1594) | 19 | 3309 (655) | 1809 (655) |
| April | 27 | 3305 (901) | 1805 (901) | 14 | 3192 (605) | 1692 (605) |
| May | 19 | 3176 (627) | 1676 (627) | 20 | 3029 (443) | 1529 (443) |
| June | 58 | 3319 (881) | 1819 (881) | 47 | 3260 (759) | 1760 (759) |
| July | 44 | 3134 (467) | 1634 (467) | 27 | 3126 (667) | 1626 (667) |
| August | 21 | 3611 (1144) | 2111 (1144) | 14 | 3242 (1746) | 1742 (1746) |
| September | 28 | 3413 (746) | 1913 (746) | 16 | 2964 (415) | 1464 (415) |
| October | 36 | 3416 (639) | 1916 (639) | 23 | 3395 (708) | 1895 (708) |
| November | 57 | 3503 (866) | 2003 (866) | 25 | 3575 (549) | 2075 (549) |
| December | 58 | 3339 (709) | 1839 (709) | 16 | 3318 (750) | 1818 (750) |
| *Timing of admission (3-month windows)* |  |  |  |  |  |  |
| 2017 (October to December) | 27 | 2957 (463) | 1457 (463) | 2 | 2975 (106) | 1475 (106) |
| 2018 (January to March) | 30 | 3387 (1356) | 1887 (1356) | 16 | 3232 (695) | 1732 (695) |
| 2018 (April to June) | 90 | 3306 (870) | 1806 (870) | 67 | 3157 (667) | 1657 (667) |
| 2018 (July to September) | 45 | 3461 (951) | 1961 (951) | 35 | 3001 (654) | 1501 (654) |
| 2018 (October to December) | 49 | 3321 (615) | 1821 (615) | 18 | 3184 (693) | 1684 (693) |
| 2019 (January to March) | 21 | 3165 (550) | 1665 (550) | 10 | 3083 (562) | 1583 (562) |
| 2019 (April to June) | 16 | 3209 (617) | 1709 (617) | 16 | 3366 (647) | 1866 (647) |
| 2019 (July to September) | 46 | 3189 (522) | 1689 (522) | 20 | 3263 (1421) | 1763 (1421) |
| 2019 (October to December) | 75 | 3650 (836) | 2150 (836) | 44 | 3574 (626) | 2074 (626) |
| 2020 (January to February) | 32 | 3259 (702) | 1759 (702) | 28 | 3310 (742) | 1810 (742) |

Footnote: Unit of costing – Cost per patient per day in LKR, Metabolic comorbidities include diabetes mellitus, hypertension, hyperlipidaemia, past history of major cardiovascular events or a combination of these.
