# Supplementary material for: Direct costs of managing in-ward dengue patients in Sri Lanka: A prospective study
Source: PLoS One. 2021 Oct 8;16(10):e0258388. doi: 10.1371/journal.pone.0258388 (PMC8500425; doi:10.1371/journal.pone.0258388)
Supplement: S2 Table — (DOCX) [file pone.0258388.s002.docx]

**Supplementary Table 2.** Mean differences of costs within DF and NDF patient groups (the first subgroup in each category is the comparator, Unit– cost per patient per day in LKR)

| Characteristic | Dengue fever | | Non-dengue fever | |
| --- | --- | --- | --- | --- |
|  | Mean difference in LKR | P value | Mean difference in LKR | P value |
| Gender |  |  |  |  |
| Male | - | - | - | - |
| Female | -68 | 0.4160 | 48 | 0.6389 |
| Age group |  |  |  |  |
| Age<=20 years | - | - | - | - |
| Age 21-30 years | -9 | 0.9362 | 18 | 0.9146 |
| Age 31-40 years | -40 | 0.7487 | 108 | 0.4054 |
| Age 41-50 years | 79 | 0.5447 | 196 | 0.2380 |
| Age 51-60 years | -92 | 0.5556 | 17 | 0.9216 |
| Age 61-70 years | 201 | 0.3683 | 84 | 0.6088 |
| Age >=71 years | -296 | 0.5833 | 69 | 0.7696 |
| Metabolic comorbidities |  |  |  |  |
| Yes | - | - | - | - |
| No | -21 | 0.8434 | -145 | 0.1746 |
| Plasma leakage |  |  |  |  |
| Yes | - | - | - | - |
| No | -40 | 0.6447 | NA | NA |
| Severe dengue |  |  |  |  |
| Yes | - | - | - | - |
| No | 86 | 0.6112 | NA | NA |
| Serotype |  |  |  |  |
| DENV-2 | - | - | - | - |
| Others | -17 | 0.8607 | NA | NA |
| Month of admission |  |  |  |  |
| January | - | - | - | - |
| February | 148 | 0.4360 | -368 | 0.1532 |
| March | -199 | 0.4650 | -225 | 0.2752 |
| April | -43 | 0.8100 | -108 | 0.6206 |
| May | 86 | 0.6207 | 55 | 0.7525 |
| June | -57 | 0.7122 | -176 | 0.3463 |
| July | 128 | 0.2789 | -41 | 0.8261 |
| August | -349 | 0.1119 | -157 | 0.6976 |
| September | -151 | 0.3548 | 121 | 0.5194 |
| October | -154 | 0.2790 | -310 | 0.1299 |
| November | -241 | 0.1155 | -491 | 0.0070* |
| December | -77 | 0.5661 | -233 | 0.3093 |
| Year of admission |  |  |  |  |
| 2018 | - | - |  |  |
| 2019 | -53 | 0.5497 | -284 | 0.0060* |

Reference category for comparison, Unit of costing – cost per patient per day in LKR

* Statistically significant with the p value of <0.05

** Statistically significant with the Bonferroni adjusted p value of <0.007
