# Supplementary material for: Direct costs of managing in-ward dengue patients in Sri Lanka: A prospective study
Source: PLoS One. 2021 Oct 8;16(10):e0258388. doi: 10.1371/journal.pone.0258388 (PMC8500425; doi:10.1371/journal.pone.0258388)
Supplement: S3 Table — (DOCX) [file pone.0258388.s003.docx]

**Supplementary Table 3.** Mean differences of costs across DF and NDF patient groups (a positive mean difference indicates a higher cost in DF group, Unit– cost per patient per day in LKR)

| Characteristic | Dengue and Non-dengue fever | |
| --- | --- | --- |
|  | Mean difference in LKR | P value |
| Gender |  |  |
| Male | 55 | 0.4610 |
| Female | 171 | 0.1440 |
| Age group |  |  |
| Age<=20 years | 23 | 0.8603 |
| Age 21-30 years | 50 | 0.7142 |
| Age 31-40 years | 172 | 0.2315 |
| Age 41-50 years | 141 | 0.3942 |
| Age 51-60 years | 132 | 0.5208 |
| Age 61-70 years | -93 | 0.6738 |
| Age >=71 years | 389 | 0.3600 |
| Metabolic comorbidities |  |  |
| Yes | 181 | 0.1056 |
| No | 57 | 0.4461 |
| Month of admission |  |  |
| January | 178 | 0.2820 |
| February | -338 | 0.2417 |
| March | 152 | 0.7018 |
| April | 113 | 0.6757 |
| May | 147 | 0.4022 |
| June | 59 | 0.7184 |
| July | 8 | 0.9547 |
| August | 369 | 0.4541 |
| September | 449 | 0.0322* |
| October | 21 | 0.9054 |
| November | -72 | 0.7028 |
| December | 21 | 0.9180 |
| Year of admission |  |  |
| 2017 (October to December) | -18 | 0.9584 |
| 2018 (January to March) | 155 | 0.6716 |
| 2018 (April to June) | 149 | 0.2453 |
| 2018 (July to September) | 461 | 0.0166* |
| 2018 (October to December) | 137 | 0.4393 |
| 2019 (January to March) | 82 | 0.7041 |
| 2019 (April to June) | -157 | 0.4875 |
| 2019 (July to September) | - 74 | 0.7564 |
| 2019 (October to December) | 75 | 0.6061 |
| 2020 (January to February) | -52 | 0.7836 |

* Statistically significant with the p value of <0.05

** Statistically significant with the Bonferroni adjusted p value of <0.004 (month of admission), p value of <0.005 (year of admission)
