# Supplementary material for: Direct costs of managing in-ward dengue patients in Sri Lanka: A prospective study
Source: PLoS One. 2021 Oct 8;16(10):e0258388. doi: 10.1371/journal.pone.0258388 (PMC8500425; doi:10.1371/journal.pone.0258388)
Supplement: S4 Table — (DOCX) [file pone.0258388.s004.docx]

**Supplementary Table 4.** Investigation numbers and associated costs in LKR during the 29-month assessment period

| Investigation | Cost per unit in LKR | Number of tests | Total investigation cost in LKR |
| --- | --- | --- | --- |
| Full blood count | 250 | 5840 | 1,460,000 |
| AST | 100 | 1742 | 174,200 |
| ALT | 100 | 1742 | 174,200 |
| Serum electrolyte | 250 | 1488 | 372,000 |
| Serum creatinine | 150 | 1476 | 221,400 |
| CRP | 200 | 1311 | 262,200 |
| Ultrasound scan | 450 | 1085 | 488,250 |
| Bilirubin | 150 | 781 | 117,150 |
| NS 1 test | 780 | 687 | 535,860 |
| UFR | 90 | 201 | 18,090 |
| Creatine Kinase (CPK) | 300 | 203 | 60,900 |
| ALP | 150 | 137 | 20,550 |
| ESR | 50 | 111 | 5,550 |
| X-rays | 150 | 103 | 15,450 |
| ECG | 50 | 87 | 4,350 |
| PT/INR | 100 | 81 | 8,100 |
| Albumin and globulin | 125 | 69 | 8,625 |
| Serum protein | 125 | 63 | 7,875 |
| APTT | 150 | 58 | 8,700 |
| Urine culture + ABST | 350 | 35 | 12,250 |
| Amylase | 200 | 35 | 7,000 |
| Serum urea | 100 | 34 | 3,400 |
| Gamma GT | 150 | 29 | 4,350 |
| Serum calcium | 150 | 27 | 4,050 |
| Blood culture +ABST | 350 | 24 | 8,400 |
| Plasma glucose | 125 | 10 | 1,250 |
| Troponin | 800 | 9 | 7,200 |
| Sputum culture + ABST | 350 | 8 | 2,800 |
| Echocardiography | 2000 | 7 | 14,000 |
| IgG test | 400 | 5 | 2,000 |
| IgM test | 400 | 4 | 1,600 |
| Stool culture +ABST | 350 | 3 | 1,050 |
| Total cholesterol | 150 | 3 | 450 |

Footnote: ABST: Antibiotic Sensitivity Test, ALP: Alkaline Phosphatase, ALT: Alanine
