# Supplementary material for: Direct costs of managing in-ward dengue patients in Sri Lanka: A prospective study
Source: PLoS One. 2021 Oct 8;16(10):e0258388. doi: 10.1371/journal.pone.0258388 (PMC8500425; doi:10.1371/journal.pone.0258388)
Supplement: S5 Table — (DOCX) [file pone.0258388.s005.docx]

**Supplementary Table 5**. Recalculated cost for non-dengue fever patients based on several hypothetical scenarios assuming RT-PCR based exclusion of dengue diagnosis was available on admission.

| **Scenario** | **Patients in Q1*** | **Patients in Q2, Q3*** | **Patients in Q4*** | **Average cost per patient in USD (SD)** | **Average cost per patient in LKR (SD)**** |
| --- | --- | --- | --- | --- | --- |
| 1 | FBC+RT_PCR+NS1 | FBC+RT_PCR+NS1 | RT_PCR+ NS1+ Investigation costs during stay + base cost | 35.74 (18.52) | 6627 (3434) |
| 2 | FBC+RT_PCR+NS1 | FBC+RT_PCR+NS1+20% IgM | RT_PCR+ NS1+ Investigation costs during stay + base cost | 36.05 (18.42) | 6686 (3415) |
| 3 | FBC+RT_PCR+NS1 | FBC+RT_PCR+NS1+50% IgM | RT_PCR+ NS1+ Investigation costs during stay + base cost | 36.53 (18.25) | 6773 (3384) |
| 4 | FBC+RT_PCR+NS1 | FBC+RT_PCR+NS1 | RT_PCR+ NS1+ Investigation costs during stay + base cost | 78.92 (18.52) | 14635 (3434) |
| 5 | FBC+RT_PCR+NS1 | FBC+RT_PCR+NS1+20% IgM | RT_PCR+ NS1+ Investigation costs during stay + base cost | 79.24 (18.42) | 14694 (3415) |
| 6 | FBC+RT_PCR+NS1 | FBC+RT_PCR+NS1+50% IgM | RT_PCR+ NS1+ Investigation costs during stay + base cost | 79.71 (18.25) | 14781 (3384) |

*Patients are divided into quartiles based on a symptom score (see methods) on admission with Q1 having least symptomatic and Q4 having the most symptomatic patients.

**Exchange rate: USD 1 = LKR 185.43 (average exchange rate in 2020)

Scenario 1: All patients have RT-PCR (at a subsidised rate of USD 22.4) and an NS1 test on admission. Patients in Q1, Q2 and Q3 are discharged home without any hospital stay and none of them return follow up. Patients in Q4 remain admitted incur the same costs as they actually did in real life.

Scenario 2: As in Scenario 1 but assumes 20% of patients in Q2 and Q3 will return for a dengue IgM test

Scenario 3: As in Scenario 1 but assumes 50% of patients in Q2 and Q3 will return for a dengue IgM test

Scenarios 4-6: Corresponding to scenarios 1-3 respectively, but assumes a commercial rate for RT-PCR (USD 65.6)
